# P15 UK Antimicrobial Registry Study: progress to date

**DOI:** 10.1093/jacamr/dlad143.019

**Published:** 2024-01-03

**Authors:** Rebecca Parr, Jay Woods, Gareth T Jones, Gary J Macfarlane, Jacqueline Sneddon, Laura J Moir, The UK Antimicrobial Registry Steering Committee, David Jenkins, Nicholas Brown, Andrew Seaton, Jonathan Sandoe, Christopher Longshaw, Fran Garraghan

**Affiliations:** Epidemiology Group, School of Medicine, Medical Sciences and Nutrition, University of Aberdeen, Aberdeen, UK; Epidemiology Group, School of Medicine, Medical Sciences and Nutrition, University of Aberdeen, Aberdeen, UK; Epidemiology Group, School of Medicine, Medical Sciences and Nutrition, University of Aberdeen, Aberdeen, UK; Epidemiology Group, School of Medicine, Medical Sciences and Nutrition, University of Aberdeen, Aberdeen, UK; British Society for Antimicrobial Chemotherapy, Birmingham, UK; Epidemiology Group, School of Medicine, Medical Sciences and Nutrition, University of Aberdeen, Aberdeen, UK

## Abstract

**Background:**

Effective antimicrobial stewardship is key in the fight against antimicrobial resistance, a key element of which is the surveillance of antimicrobial use and antimicrobial resistance. In addition to quantitative analysis of antibiotic use trends it is also important to understand qualitative aspects of how antimicrobials are used in routine clinical settings.

**Objectives:**

The UK Antimicrobial Registry (UKAR) Study, a 5 year collaboration between BSAC and the University of Aberdeen, aims to determine how recently licensed antimicrobial agents are being used, their effectiveness and safety. The study is included in the NIHR portfolio in England with similar arrangements in Scotland and Wales. Here, we present brief information on the first few months of participant recruitment.

**Methods:**

The study commenced recruiting patients prescribed selected antibiotics in May 2023. The intention was to commence with a small number of pilot hospital sites then to expand to a larger number of hospitals. Adult patients are potentially eligible if they have been prescribed any drug on the eligible drug list within their current stay in hospital unless the drug is actively withdrawn after less than 24 h. Data are collected from the medical records of participants who have given informed consent. Clinical data, including infection, microbiology susceptibility testing, eligible drug and dosing regimens are collected as well as prior and concurrent antibiotic therapy.

**Results:**

As of November 2023, the study is open to recruitment in nine sites across the UK. There are a further 22 sites across the UK in study set-up and a further 15 sites who have submitted an expression of interest to join the registry. Fifty-two patients have been recruited as of November 2023. Five of the 11 study drugs were prescribed; the majority of the participants (*N*=29) were prescribed dalbavancin, the remainder received various Gram-negative agents (Table 1). Study drugs were used to treat bone and joint infections (33%) and lower respiratory tract infections (33%), with other disease areas including skin and subcutaneous tissue infections and urinary tract infections.

**Conclusions:**

A key objective for UKAR pilot sites was to verify that the data collection process was feasible and address any issues around clarity of responses. Very few issues were identified, and all were resolved quickly. Early data show that participants are most likely to be female with a median age above 60 years. There is also, currently, a preponderance of patients commencing dalbavancin, although the study is still in its infancy. As more sites recruit participants to the UKAR Study, more detailed analyses can be performed on the clinical usage and outcomes being achieved. Also, follow-up data including clinical outcomes and adverse events will be collected 56 days after commencing therapy, and 28 days after last administration of an eligible study drug. In addition, clinical outcomes and relapse of the initial infection will be recorded at 6 and 12 months.

